# Association of glycemic variability and hypoglycemia with distal symmetrical polyneuropathy in adults with type 1 diabetes

**DOI:** 10.1038/s41598-021-02258-3

**Published:** 2021-11-24

**Authors:** Ziyang Shen, Hemin Jiang, Rong Huang, Yunting Zhou, Qian Li, Jianhua Ma

**Affiliations:** 1 Department of Endocrinology, Nanjing First Hospital, Nanjing Medical University, Nanjing, 210012 China; 2 Department of Endocrinology, The First Affiliated Hospital of Nanjing Medical University, Nanjing, 210012 China

**Keywords:** Endocrinology, Neurology

## Abstract

Previous studies exploring the influence of glycemic variability (GV) on the pathogenesis of distal symmetrical polyneuropathy (DSPN) in type 1 diabetes (T1DM) produced conflicting results. The aim of this study was to assess the relationship between GV and DSPN in T1DM. Adults with T1DM were included in this cross-sectional study and asked to undergo 3-day CGM. GV quantified by coefficient of variation (CV) and mean amplitude of glucose excursions (MAGE) were obtained from CGM. Clinical characteristics and biochemical assessments were collected for analysis. The study comprised 152 T1DM patients (53.9% males) with mean age of 44.2 year. Higher levels of age and duration of diabetes and lower levels of total cholesterol, LDL, fasting C-peptide and postprandial C-peptide were observed in DSPN subjects. DSPN groups displayed a higher blood glucose between 00:00 and 12:59 according to the CGM profile. Higher MAGE and CV were associated with increased risk of DSPN in the fully adjusted model. Meanwhile, a significant association between measurements of hypoglycemia, especially nocturnal hypoglycemia, and DSPN was found after multiple tests. CGM parameters describing the glycemic variability and hypoglycemia were potential risk factors for DSPN in adults with T1DM.

## Introduction

As one of the most prevalent chronic complications of diabetes mellitus, distal symmetrical polyneuropathy (DSPN) would affect about 50 million people worldwide by the year of 2030^1,2^. Typically, DSPN patients suffer from pain and hypersensitivity followed by mechanical and thermal hypoalgesia, which increase the risk of foot ulceration and amputation^3^. These symptoms result in reduced quality of life and economic burden significantly^4^. Currently, there are no approved disease modifying therapies for DSPN in addition to intensive glycemic control^5^.

Glycemic variability (GV) is a term describing glycemic fluctuations within a given time interval and has been used as an important metric to evaluate glucose homoeostasis in clinical practice^6^. Increasing clinical and laboratory evidence suggests that GV is associated with DSPN independent of mean glucose in type 2 diabetes (T2DM), by causing Schwann cells apoptosis and oxidative stress^7–10^. Unfortunately, data on the relationship between GV and DSPN in type 1 diabetes (T1DM) is inconsistent and scarce. Several studies suggest GV as an independent risk factor of DSPN and peripheral nerve dysfunction^11,12^. Conversely, following Diabetes Control and Complications Trial (DCCT) and other studies deny the association of GV with DSPN^13–15^. However, most previous studies are limited to a small sample size, self-reporting blood glucose profiles and evaluation of GV by measures of Glycated hemoglobin (HbA1c), which may be an insufficient tool to assess GV^16^. Continuous glucose monitoring (CGM) has been recommended as the primary assessment of GV. Moreover, recent studies demonstrate the importance of hypoglycemia on impaired autonomic cardiovascular function in patients with T1DM^17^. T1DM patients suffer from great risk of hypoglycemia due to exogenous injection of insulin and impaired glucagon and the adrenalin responses. Paralysis following hypoglycemia in T1DM patients has been documented in the literature^18^. Hypoglycemic coma is independently related to neuropathy^19^. Thus, the influence of CGM-defined degree of hypoglycemia on DSPN should also be explored.

Our previous studies have indicated the association of GV with central nervous system damage and diabetic complications, such as diabetic nephropathy based on the date from CGM^20,21^. The aim of current study was to explore the relative contribution of each internationally standardized CGM metric to DSPN in T1DM patients.

## Methods

### Study population

This cross-sectional study was conducted at Department of Endocrinology, Nanjing First Hospital from July 2016 to May 2019. Individuals older than 20 years with T1DM who satisfied the American Diabetes Association diagnostic criteria were recruited^22^. All included subjects should take insulin treatment within 6 months after diagnosis and met the criteria of fasting C-peptide < 0.6 ng/mL or more than one positive autoantibody. Participants were excluded if they: 1) suffered other peripheral neurological illness or major medical illness, such as cancer, sever liver dysfunction; 2) could not wear the CGM. DSPN was diagnosed with the Michigan Neuropathy Screening Instrument (MNSI) examination score > 2 using the second component of the MNSI^23^. Ultimately, 152 participants were enrolled in our study and signed informed consent. Continuous subcutaneous insulin infusion was used during the period of hospitalization for a better control of blood glucose. This study was approved by the research ethics committees of the Nanjing First Hospital, Nanjing Medical University and conducted according to the principles of the Declaration of Helsinki.

### Clinical and biochemical assessments

All anthropometric parameters such as age, height, weight, hip and waist circumference, heart rate and blood pressure were recorded upon entry into the study. Fasting venous blood samples drawn after overnight were used to measure biochemical biomarkers including HbA1c (using high performance liquid chromatography on a Tosoh G7), C-peptide (using Cobas e411), lipid profiles including triglycerides, high-density lipoprotein (HDL), low-density lipoprotein (LDL) and total cholesterol (using standard enzymatic colorimetry techniques on a Vitros 5600), fasting plasma glucose (FPG, using the standard enzymatic method) and serum creatinine.

### CGM parameters

The CGM sensor (Medtronic Incorporated, Northridge, Minnesota, USA) was inserted into anterior abdominal skin of each subject for at least 3 days. Capillary finger blood glucose was measured four times daily for calibration. To avoid the bias caused by insertion and removal of the sensor, only glycemic profiles obtained from 0:00 to 24:00 on day 2 were adopted for further analysis. Mean glucose, mean amplitude of glycemic excursions (MAGE), coefficient of variation (CV) and glucose management indicator (GMI) were calculated to evaluate glucose fluctuation. Low blood glucose index (LBGI) was calculated using the Easy GV software^24^. The time in hypoglycemia or hyperglycemia and area under the curve (AUC) of hypoglycemia or hyperglycemia were also obtained. Hypoglycemia was classified as previously described: level 1 for glucose 3.0–3.9 mmol/L or level 2 for < 3.9 mmol/L. Time frame for nocturnal hypoglycemia was set from 0 to 6 am when individuals were asleep. Hyperglycemia was categorized as level 1 for 10–13.9 mmol/L or level 2 for > 13.9 mmol/L^25^.

### Statistical analysis

Patient characteristics are presented as means ± standard error (SE) for continuous variables, or as median (interquartile range) for skewed distributions. Student's unpaired t‐test or Mann‐Whitney U‐test were performed to compare differences between subjects with and without DSPN. Categorical variables were analyzed by the Chi-square test. Univariate and multivariate logistic regression analyses were adopted to assess the effect of GV parameters and other risk factors on DSPN. ALL statistical analyses were carried out using the SPSS 23.0 software (SPSS, Science, Chicago, Illinois). Statistical significance was defined as a two-tailed P-value < 0.05.

## Results

### Patient characteristics

As shown in Table 1, 152 patients with T1DM were included in this study with a mean age of 44.2 ± 13.9 years, average HbA1c of 9.5 ± 2.2% and median duration of diabetes of 7.0 years. In total, 41 subjects were diagnosed with DSPN. A significantly higher levels of age and duration of diabetes and lower levels of total cholesterol, LDL, fasting C-peptide and postprandial C-peptide were observed in DSPN patients. Whereas, we did not find difference of male gender, BMI, waist-hip ratio, current smoker ratio, systolic/diastolic blood pressure, heart rate, triglycerides, HDL, HbA1c, estimated glomerular filtration (eGFR), family history and medication between the two groups.

**Table 1:** Baseline characteristics of enrolled subjects with type 1 diabetes.

Variables	Total subjects (n = 152)	No DSPN (n = 111)	DSPN (n = 41)	P value
Age (years)	44.2 ± 13.9	42.7 ± 13.7	48.4 ± 13.7	0.022
Sex, male (%)	82 (53.9)	58 (52.3)	24 (58.5)	0.490
Body mass index (kg/m^2^)	20.9 ± 2.9	21.0 ± 3.0	20.8 ± 2.4	0.841
Waist-hip ratio	0.9 ± 0.1	0.9 ± 0.2	0.9 ± 0.1	0.401
Current smoker, n (%)	21 (13.8)	15 (13.5)	6 (14.6)	0.295
Systolic blood pressure (mmHg)	119.4 ± 18.5	118.8 ± 17.3	121.1 ± 21.6	0.481
Diastolic blood pressure (mmHg)	75.6 ± 9.6	75.7 ± 8.7	75.0 ± 11.9	0.697
Heart rate (bpm)	79.7 ± 12.7	79.0 ± 12.8	81.6 ± 12.2	0.261
Diabetes duration (years)	7.0 (2.0–10.0)	5.0 (1.0–10.0)	10.0 (4.5–12.5)	0.002
Total cholesterol (mmol/L)	4.4 ± 1.0	4.5 ± 1.1	4.2 ± 0.8	0.042
Triglycerides (mmol/L)	0.8 (0.6–1.2)	0.8 (0.6–1.2)	0.8 (0.6–1.6)	0.512
LDL (mmol/L)	1.9 (1.4–2.3)	2.0 (1.5–2.4)	1.8 (1.3–2.2)	0.085
HDL (mmol/L)	1.4 (1.2–1.7)	1.4 (1.2–1.7)	1.4 (1.2–1.9)	0.558
Fasting plasma glucose (mmol/L)	10.8 ± 5.6	10.5 ± 5.7	11.7 ± 5.1	0.281
Fasting C-peptide (ng/mL)	0.0 (0.0–0.2)	0.0 (0.0–0.2)	0.0 (0.0–0.1)	0.109
Postprandial C-peptide (ng/mL)	0.0 (0.0–0.4)	0.1 (0.0–0.4)	0.0 (0.0–0.2)	0.082
Hemoglobin A1c (%)	9.5 ± 2.2	9.5 ± 2.3	9.8 ± 2.0	0.413
Family history, Yes(%)	28 (18.4)	22 (19.8)	6 (14.6)	0.464
eGFR (ml/min/1.73m^2^)	132.0 ± 43.9	132.7 ± 43.6	130.0 ± 45.1	0.736
**Medication**				
Insulin treatment n (%)	152 (100.0)	111 (100.0)	41 (100.0)	0.115
Metformin n (%)	9 (5.9)	4 (3.6)	5 (12.2)	
Other glucose-lowering drugs n (%)	4 (2.6)	4 (3.6)	0 (0.0)	

HDL, high-density lipoprotein; LDL, low-density lipoprotein; eGFR, estimated glomerular filtration.

### CGM parameters between groups

**Figure 1:**
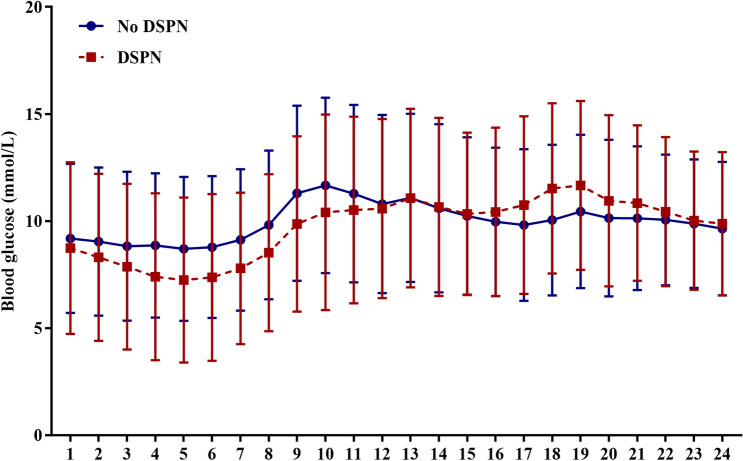
Comparison of hourly glucose concentrations between subjects with and without DSPN.

CGM profiles from day 2 were analyzed hourly to evaluate the glycemic variability. DSPN groups owned a higher blood glucose than those without DSPN between 00:00 and 12:59 (Fig. 1). Detailed CGM parameters were summarized in Table 2. Although there was no difference in mean glucose between individuals with and without DSPN, significant higher GV parameters such as CV and MAGE were observed in DSPN patients. Interestingly, we also observed significant upregulated percent time and AUC of hypoglycemia including level 1 and level 2 hypoglycemia, especially the nocturnal hypoglycemia in the DSPN subjects. Whereas, the percent time and AUC of hyperglycemia displayed an increased trend but failed to reach the significance.

**Table 2:** CGM parameters of T1DM patients according to presence of DSPN.

Variables	No DSPN (n = 111)	DSPN (n = 41)	P value
Mean glucose (mmol/L)	10.0 ± 2.6	9.7 ± 2.6	0.586
**Parameters of glycemic variability**			
MAGE	5.8 ± 3.1	7.2 ± 3.3	0.018
Coefficient of variance (%)	24.8 ± 11.6	32.9 ± 11.5	< 0.001
LBGI	0.2 (0.0–1.0)	0.9 (0.0–1.0)	0.003
**Parameters of hypoglycemia**			
**Percent time in hypoglycemia**			
Level 1 (3.0–3.9 mmol/L)	0.0 (0.0–0.0)	0.0 (0.0–0.9)	0.002
Level 2 (< 3.0 mmol/L)	0.0 (0.0–0.0)	0.0 (0.0–7.4)	0.005
Total (< 3.9 mmol/L)	0.0 (0.0–0.0)	0.0 (0.0–9.2)	0.002
AUC hypoglycemia (mmol/L * min)			
Level 1 (3.0–3.9 mmol/L)	0.0 (0.0–0.0)	0.0 (0.0–16.4)	0.003
Level 2 (< 3.0 mmol/L)	0.0 (0.0–0.0)	0.0 (0.0–0.2)	0.005
Total (< 3.9 mmol/L)	0.0 (0.0–0.0)	0.0 (0.0–14.8)	0.003
**Percent time in nocturnal hypoglycemia**			
Level 1 (3.0–3.9 mmol/L)	0.0 (0.0–0.0)	0.0 (0.0–3.8)	< 0.001
Level 2 (< 3.0 mmol/L)	0.0 (0.0–0.0)	0.0 (0.0–0.0)	0.002
Total (< 3.9 mmol/L)	0.0 (0.0–0.0)	0.0 (0.0–6.9)	< 0.001
AUC nocturnal hypoglycemia (mmol/L * min)			
Level 1 (3.0–3.9 mmol/L)	0.0 (0.0–0.0)	0.0 (0.0–9.9)	< 0.001
Level 2 (< 3.0 mmol/L)	0.0 (0.0–0.0)	0.0 (0.0–0.0)	0.002
Total (< 3.9 mmol/L)	0.0 (0.0–0.0)	0.0 (0.0–10.9)	< 0.001
**Parameters of hyperglycemia**			
**Percent time in hyperglycemia**			
Level 1 (10–13.9 mmol/L)	23.3 (11.1–39.6)	20.1 (12.5–28.5)	0.256
Level 2 (> 13.9 mmol/L)	5.6 (0.0–23.3)	13.2 (0.7–25.9)	0.185
Total (> 10.0 mmol/L)	39.2 (16.3–62.5)	40.3 (20.8–49.8)	0.853
AUC hyperglycemia (mmol/L * min)			
Level 1 (10–13.9 mmol/L)	229.7 (55.6–499.4)	265.0 (87.2–447.8)	0.572
Level 2 (> 13.9 mmol/L)	9.9 (0.0–158.6)	55.6 (0.5–180.0)	0.159
Total (> 10.0 mmol/L)	260.5 (55.6–650.9)	340.9 (88.5–612.1)	0.433

CGM, continuous glucose monitoring; MAGE, mean amplitude of glycemic excursion; AUC, area under the curve.

### Number of hypoglycemic episodes between groups

Among the 34 patients who experienced hypoglycemic episodes at least one time during the 24 h of CGM, 16 (47.1%) subjects suffered from DSPN. Episodes of prolonged hypoglycemia were also more frequent in subjects with DSPN (24.4% vs. 6.3%). As for the nocturnal episodes of hypoglycemia, we also observed a higher proportion of nocturnal hypoglycemia (36.6% vs. 10.0%) as well as nocturnal prolonged hypoglycemia (2.7% vs. 19.5%) in DSPN groups when compared with control group.

### Associations between CGM parameters and DSPN

**Figure 2:**
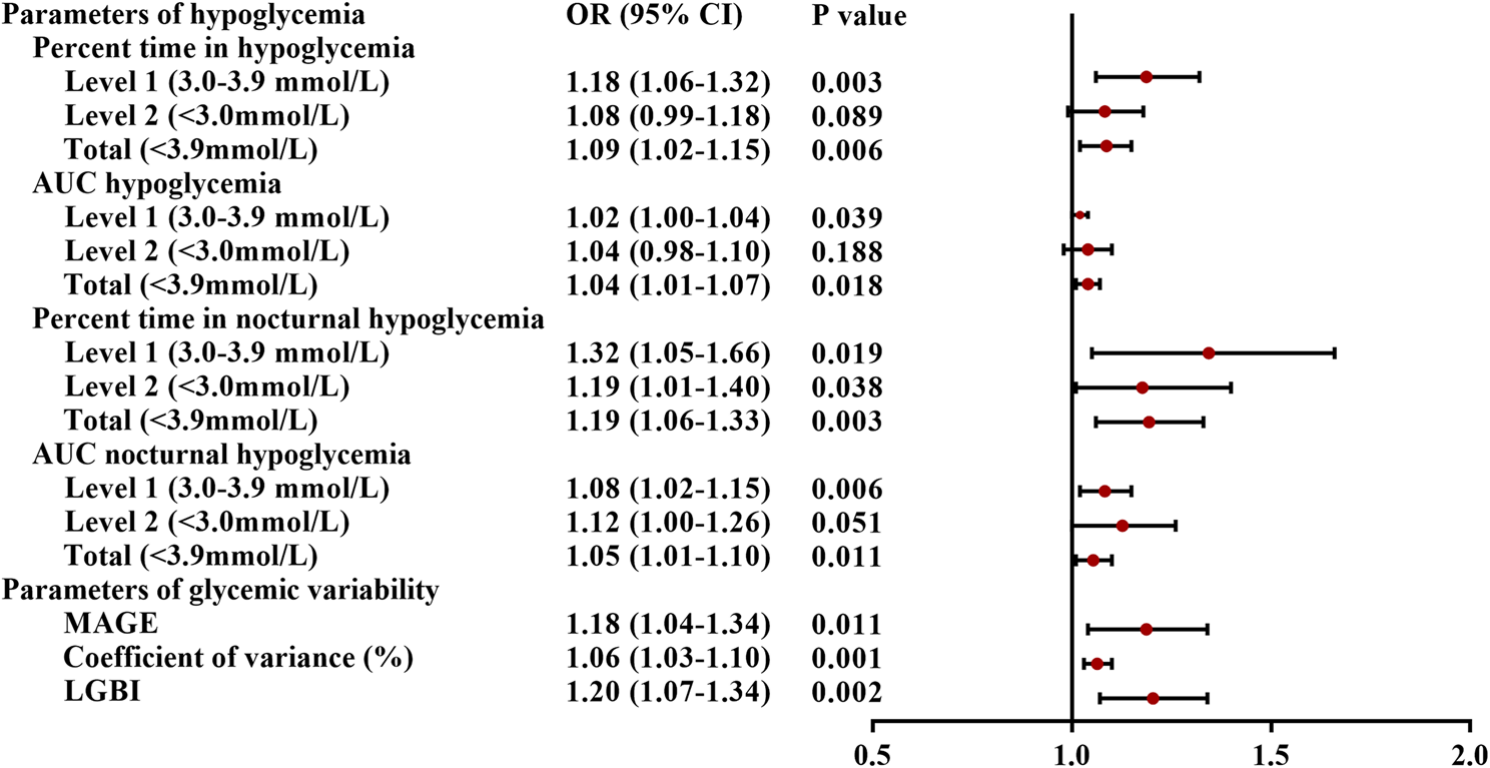
Odds ratios and 95% confidence intervals comparing the risk of CGM parameters for subjects with and without DSPN.

Univariate and multivariate logistic regression analyses were performed for each CGM parameter to assess the relationship between CGM parameters and DSPN. Clinical variables other than CGM parameters showed significant difference between groups and clinical relevance as reported by previous studies, such as mean glucose, age, durations of diabetes, total cholesterol, LDL, HDL, fasting C-peptide and postprandial C-peptide, and HbA1c were included in the multivariate logistic regression analyses. GV and parameters about hypoglycemia reached statistical significance in the univariate model (Supplementary Table 1). As shown in Fig. 2, increased values of CV, MAGE and LBGI were independent risk factors for DSPN patient according the multivariate models. Moreover, both percent time in hypoglycemia and AUC of hypoglycemia were associated with increased risk of DSPN after adjustment for mean glucose and other potential risk factors. However, percent time of level 2 hypoglycemia and AUC of level 2 hypoglycemia and nocturnal hypoglycemia lost the significant difference in the multivariate models.

## Discussion

In this study, we found that CGM parameters describing GV were strongly associated with DSPN in T1DM patients. Moreover, our results suggested the hypoglycemia as a risk factor of DSPN.

Currently, only few previous studies questioned the association of GV and DSPN in T1DM and presented conflicting results. Miho et al. found a significantly positive association of MAGE with medial plantar neuropathy after adjustment for clinical background based on the data of seven days CGM^26^. However, more than half T2DM patients were included and analyzed as a whole in this study. Since GV have be well documented as a risk factor of DSPN in T2DM^9^, conclusions from this study might not be simply extrapolated to population with T1DM. Another study investigating the relationship between GV and peripheral nerve function in seventeen T1DM patients demonstrated a strong positive correlation of MAGE with impaired motor and sensory axonal function independent of acute glucose levels. But patients recruited in research conducted by Krishnan et al. had no sensory or motor symptoms and had normal nerve conduction studies^12^. Thus, GV might serve as a risk factor of axonal dysfunction. But, it was not enough power to directly link GV to DSPN in T1DM. Similarly, reducing GV with continuous subcutaneous insulin infusion could reverse the axonal dysfunction in T1DM. However, there was no evidence for the improvement of clinical symptoms^27^. To the best knowledge, our study demonstrated the positive association of GV with DSPN in T1DM individuals based on the CGM profiles for the first time. Whereas, conflicting results documented by previous studies should also be noted^13–15,28^. Moreover, Sohaib et al. explored the long-term GV by standard deviation of all HbA1c measurements and indicated tight association of GV with retinopathy, early nephropathy and cardiac autonomic neuropathy, but not peripheral neuropathy^14^. From a theoretical standpoint, GV could increase oxidative stress, induce inflammatory cytokines secretion and promote systemic inflammation which might contribute to the development of DSPN^29^. But current evidences could not confer a solid conclusion for the relationship between GV and DSPN. Further research is required to fully explore the role of GV in DSPN and elucidate the precise mechanisms mediating the association.

Early optimization of glucose control is recommended to prevent or delay DSPN in T1DM^1^. On the other hand, intensive insulin treatment might increase risk of hypoglycemia, especially in T1DM patients who had poor islet function. Our study indicated that parameters quantifying the hypoglycemia significantly correlated with DSPN in T1DM. Peripheral nervous system damage associated with hypoglycemia has been reported as early as 70 years ago^18^. Long-term mild hypoglycemia contributed to symmetric distal sensorimotor neuropathy and uncomfortable symptoms, such as numbness and weakness. Prolonged hypoglycemia seemed to predominantly underlies the development of motor neuropathy in contrast to the damage of sensory and autonomic nerve fibers by hyperglycemia. Given the diverse pattern of DSPN in subjects, the neuropathy observed in diabetic patients might be a combination of hyperglycemic and hypoglycemic status^30^. Studies in animals observed that hypoglycemic lesions presented as primary axonopathy followed by severe breakdown of myelin sheath while hyperglycemic caused segmental demyelination^31^. These underlying mechanisms were complex and multifactorial. Future studies were desired to clarify the exact mechanism. It was impossible to evaluate the pathologic change of peripheral nerve for each patient in clinical practice. A more frequent measurement of blood glucose by CGM might be the alternative strategy to distinguish hypoglycemic neuropathy from hyperglycemic status.

However, there are several limitations in the present study. First, this study included a small sample size. A multicenter larger sample size is desired to produce a more valid conclusion. Second, enrolled subjected were asked to wear the CGM sensors for 3 days, which might not be powerful enough to represent the daily fluctuations of blood glucose. Extension of the measuring durations was needed to confirm the findings. Moreover, as a cross-sectional study, it is impossible to evaluate the causal relationship between GV and DSPN. A prospective observational study design would be needed.

In summary, CGM parameters describing the GV such as MAGE and CV were related to DSPN in T1DM patient after adjusting for relevant risk factors. Moreover, we proposed the hypoglycemia as potential risk factors of DSPN.

## Supplementary Information

Supplementary Information.

## Abbreviations

DSPN: Distal symmetrical polyneuropathy
GV: Glycemic variability
MAGE: Mean amplitude of glycemic excursions
CV: Coefficient of variation
AUC: Area under the curve
OR: Odds ratio
BMI: Body mass index
SBP: Systolic blood pressure
HbA1c: Glycated A1c
LDL: Low-density lipoprotein
HDL: High-density lipoprotein
T2DM: Type 2 diabetes
T1DM: Type 1 diabetes
CGM: Continuous glucose monitoring
FPG: Fasting plasma glucose
eGFR: Estimated glomerular filtration
LBGI: Low blood glucose index

## References

[1] Zakin E, Abrams R, Simpson DM (2019). Diabetic neuropathy. Semin. Neurol..

[2] Fan B, Li C, Szalad A, Wang L, Pan W, Zhang R (2020). Mesenchymal stromal cell-derived exosomes ameliorate peripheral neuropathy in a mouse model of diabetes. Diabetologia.

[3] Tesfaye S, Selvarajah D, Gandhi R, Greig M, Shillo P, Fang F (2016). Diabetic peripheral neuropathy may not be as its name suggests: evidence from magnetic resonance imaging. Pain.

[4] Alleman CJ, Westerhout KY, Hensen M, Chambers C, Stoker M, Long S (2015). Humanistic and economic burden of painful diabetic peripheral neuropathy in Europe: A review of the literature. Diabetes Res. Clin. Pract..

[5] Malik RA (2020). Diabetic neuropathy: A focus on small fibres. Diab./Metab. Res. Rev..

[6] DeVries JH (2013). Glucose variability: where it is important and how to measure it. Diabetes.

[7] Yang J, Zhao Z, Yuan H, Ma X, Li Y, Wang H (2019). The mechanisms of glycemic variability accelerate diabetic central neuropathy and diabetic peripheral neuropathy in diabetic rats. Biochem. Biophys. Res. Commun..

[8] Pai YW, Lin CH, Lee IT, Chang MH (2018). Variability of fasting plasma glucose and the risk of painful diabetic peripheral neuropathy in patients with type 2 diabetes. Diab. Metab..

[9] Yang CP, Li CI, Liu CS, Lin WY, Hwang KL, Yang SY (2017). Variability of fasting plasma glucose increased risks of diabetic polyneuropathy in T2DM. Neurology.

[10] Su JB, Zhao LH, Zhang XL, Cai HL, Huang HY, Xu F (2018). HbA1c variability and diabetic peripheral neuropathy in type 2 diabetic patients. Cardiovasc. Diabetol..

[11] Jin HY, Lee KA, Park TS (2016). The impact of glycemic variability on diabetic peripheral neuropathy. Endocrine.

[12] Kwai NC, Arnold R, Poynten AM, Krishnan AV (2016). Association between glycemic variability and peripheral nerve dysfunction in type 1 diabetes. Muscle Nerve.

[13] Lachin JM, Bebu I, Bergenstal RM, Pop-Busui R, Service FJ, Zinman B (2017). Association of glycemic variability in Type 1 diabetes with progression of microvascular outcomes in the diabetes control and complications trial. Diabetes Care.

[14] Virk SA, Donaghue KC, Cho YH, Benitez-Aguirre P, Hing S, Pryke A (2016). Association between HbA1c variability and risk of microvascular complications in adolescents with Type 1 diabetes. J. Clin. Endocrinol. Metab..

[15] Christensen MMB, Hommel EE, Jørgensen ME, Fleischer J, Hansen CS (2020). Glycemic variability and diabetic neuropathy in young adults with Type 1 diabetes. Front. Endocrinol..

[16] Targets G (2018). Standards of medical care in diabetes-2018. Diabetes Care.

[17] Rao AD, Bonyhay I, Dankwa J, Baimas-George M, Kneen L, Ballatori S (2016). Baroreflex sensitivity impairment during hypoglycemia: Implications for cardiovascular control. Diabetes.

[18] Mohseni S (2001). Hypoglycemic neuropathy. Acta Neuropathol..

[19] ter Braak EW, Appelman AM, van de Laak M, Stolk RP, van Haeften TW, Erkelens DW (2000). Clinical characteristics of type 1 diabetic patients with and without severe hypoglycemia. Diabetes Care.

[20] Jin YP, Su XF, Yin GP, Xu XH, Lou JZ, Chen JJ (2015). Blood glucose fluctuations in hemodialysis patients with end stage diabetic nephropathy. J. Diabetes Complications.

[21] Xia W, Luo Y, Chen YC, Chen H, Ma J, Yin X (2020). Glucose Fluctuations Are Linked to Disrupted Brain Functional Architecture and Cognitive Impairment. J. Alzheimer's Dis.: JAD..

[22] Agiostratidou, G., Anhalt, H., Ball, D., Blonde, L., Gourgari, E., & Harriman, K.N., et al. Standardizing Clinically Meaningful Outcome Measures Beyond HbA(1c) for Type 1 Diabetes: A Consensus Report of the American Association of Clinical Endocrinologists, the American Association of Diabetes Educators, the American Diabetes Association, the Endocrine Society, JDRF International, The Leona M. and Harry B. Helmsley Charitable Trust, the Pediatric Endocrine Society, and the T1D Exchange. Diabetes care. 2017;40:1622–30. 10.2337/dc17-1624 PMC5864122 29162582

[23] Herman WH, Pop-Busui R, Braffett BH, Martin CL, Cleary PA, Albers JW (2012). Use of the Michigan Neuropathy Screening Instrument as a measure of distal symmetrical peripheral neuropathy in Type 1 diabetes: results from the Diabetes Control and Complications Trial/Epidemiology of Diabetes Interventions and Complications. Diabetic Med. J. Br. Diabetic Assoc..

[24] Perea V, Amor A, Giménez M, Blanco J, Conget I (2013). Glycemic variability measures in a group of subjects with type 1 diabetes and repeated severe and non-severe hypoglycemia. J. Diabetes Sci. Technol..

[25] Jun JE, Lee SE, Lee YB, Ahn JY, Kim G, Hur KY (2019). Continuous glucose monitoring defined glucose variability is associated with cardiovascular autonomic neuropathy in type 1 diabetes. Diabetes/Metab. Res. Rev..

[26] Akaza M, Akaza I, Kanouchi T, Sasano T, Sumi Y, Yokota T (2018). Nerve conduction study of the association between glycemic variability and diabetes neuropathy. Diabetol. Metab. Syndr..

[27] Kamel J, Loh M, Cook M, MacIsaac RJ, Roberts LJ (2020). Reducing glucose variability with continuous subcutaneous insulin infusion is associated with reversal of axonal dysfunction in type 1 diabetes mellitus. Muscle Nerve.

[28] Siegelaar SE, Kilpatrick ES, Rigby AS, Atkin SL, Hoekstra JB, Devries JH (2009). Glucose variability does not contribute to the development of peripheral and autonomic neuropathy in type 1 diabetes: data from the DCCT. Diabetologia.

[29] Sustained effect of intensive treatment of type 1 diabetes mellitus on development and progression of diabetic nephropathy: the Epidemiology of Diabetes Interventions and Complications (EDIC) study. Jama. 2003;290:2159–67. 10.1001/jama.290.16.2159 PMC2622725 14570951

[30] Mohseni S (2014). Neurologic damage in hypoglycemia. Handb. Clin. Neurol..

[31] Ozaki K, Sano T, Tsuji N, Matsuura T, Narama I (2010). Insulin-induced hypoglycemic peripheral motor neuropathy in spontaneously diabetic WBN/Kob rats. Comp. Med..

