# Supplementary material for: Association of glycemic variability and hypoglycemia with distal symmetrical polyneuropathy in adults with type 1 diabetes
Source: Sci Rep. 2021 Nov 24;11:22875. doi: 10.1038/s41598-021-02258-3 (PMC8613221; doi:10.1038/s41598-021-02258-3)
Supplement: Supplementary file 1 — Supplementary Information. [file 41598_2021_2258_MOESM1_ESM.docx]

**Association of glycemic variability and hypoglycemia with distal symmetrical polyneuropathy in adults with type 1 diabetes**

Ziyang Shen^1^, Hemin Jiang^2^, Rong Huang^1^, Yunting Zhou^1^, Qian Li^1^, Jianhua Ma^1^*

^1^ Department of Endocrinology, Nanjing First Hospital, Nanjing Medical University, Nanjing 210012, China

^2^ Department of Endocrinology, The First Affiliated Hospital of Nanjing Medical University, Nanjing 210012, China

* Correspondence author: Jianhua Ma

Department of Endocrinology, Nanjing First Hospital, Nanjing Medical University

Tel: +86-25-52887091 E-mail: majianhua196503@126.com Fax: +86-25-52887016

Supplementary Table 1 Odds ratios of CGM parameters in the univariate model

|  | Univariate model | |
| --- | --- | --- |
| Variables | OR (95% CI) | P value |
| Mean glucose (mmol/L) | 0.96 (0.84-1.11) | 0.584 |
| Parameters of glycemic variability |  |  |
| MAGE | 1.06 (1.03-1.09) | <0.001 |
| Coefficient of variance (%) | 1.14 (1.02-1.27) | 0.021 |
| LBGI | 1.16 (1.06-1.28) | 0.002 |
| Parameters of hypoglycemia |  |  |
| Percent time in hypoglycemia |  |  |
| Level 1 (3.0-3.9 mmol/L) | 1.16 (1.06-1.27) | 0.001 |
| Level 2 (<3.0 mmol/L) | 1.07 (1.00-1.16) | 0.064 |
| Total (<3.9 mmol/L) | 1.08 (1.03-1.13) | 0.003 |
| AUC hypoglycemia (mmol/L * min) |  |  |
| Level 1 (3.0-3.9 mmol/L) | 1.02 (1.00-1.04) | 0.024 |
| Level 2 (<3.0 mmol/L) | 1.04 (0.99-1.09) | 0.134 |
| Total (<3.9 mmol/L) | 1.03 (1.01-1.06) | 0.011 |
| Percent time in nocturnal hypoglycemia |  |  |
| Level 1 (3.0-3.9 mmol/L) | 1.31 (1.09-1.57) | 0.004 |
| Level 2 (<3.0 mmol/L) | 1.15 (1.01-1.32) | 0.037 |
| Total (<3.9 mmol/L) | 1.17 (1.07-1.29) | 0.001 |
| AUC nocturnal hypoglycemia (mmol/L * min) |  |  |
| Level 1 (3.0-3.9 mmol/L) | 1.08 (1.02-1.13) | 0.006 |
| Level 2 (<3.0 mmol/L) | 1.10 (1.00-1.20) | 0.061 |
| Total (<3.9 mmol/L) | 1.05 (1.01-1.09) | 0.014 |
